# Unveiling the Crucial Nexus: Mitochondrial Quality Control as a Central Driver in Metabolic Dysfunction‐Associated Steatotic Liver Disease Pathogenesis

**DOI:** 10.1111/cpr.70141

**Published:** 2025-11-09

**Authors:** Wenkai Fu, Junqi Wang, Nan Lu, Zhijiang Guo, Sang‐Bing Ong, Yong Gao, Hao Zhou, Xing Chang, Miao Meng

**Affiliations:** ^1^ The Third School of Clinical Medicine Beijing University of Traditional Chinese Medicine Beijing China; ^2^ Department of Medicine & Therapeutics, Faculty of Medicine The Chinese University of Hong Kong (CUHK) Hong Kong SAR China; ^3^ State Key Laboratory of Traditional Chinese Medicine Syndrome, Science and Technology Innovation Center Guangzhou University of Chinese Medicine Guangzhou China; ^4^ Cardiology, Senior Department of Cardiology PLA General Hospital Beijing China; ^5^ Cardiology, Guang'anmen Hospital China Academy of Chinese Medical Sciences Beijing China; ^6^ Gastroenterology, Guang'anmen Hospital China Academy of Chinese Medical Sciences Beijing China

## Abstract

Mitochondrial quality control (MQC) impairment plays a central role in driving the pathogenesis of metabolism‐associated steatotic liver disease (MASLD). Specifically, this is manifested as reduced mitophagy; increased mitochondrial fission and decreased fusion; and impaired mitochondrial biogenesis. Key pathological mechanisms of MASLD, such as hepatocyte apoptosis, pyroptosis, and ferroptosis, are activated under the influence of factors including free fatty acids (FFAs), oxidative stress, NLRP3 inflammasome activation, and gut microbiota imbalance. Meanwhile, the letter also lists novel potential therapeutic strategies targeting these pathways, including autophagy enhancers, mitochondrial dynamics regulators, biogenesis promoters, and ferroptosis inhibitors.

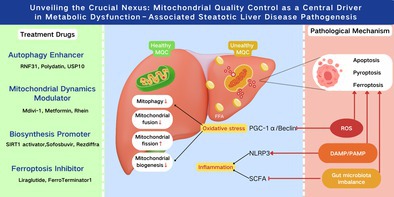

To the Editor,

1

We've been following the cutting‐edge research into mitochondrial quality control (MQC) and its role in metabolic disease pathogenesis with considerable interest. Given the skyrocketing global prevalence of metabolic dysfunction‐associated steatotic liver disease (MASLD) and the significant health burden it brings with it, getting a handle on its fundamental drivers is absolutely critical. We're writing this letter to share and chew over some fresh perspectives on MQC as a key player in how MASLD kicks off.

## 2 MQC in MASLD

### 2.1 Mitochondrial Autophagy During MASLD

Mitophagy selectively eliminates depolarized or damaged mitochondria, maintaining metabolic equilibrium through two primary pathways:

#### 2.1.1 PINK1/Parkin Pathway

**FIGURE 1:**
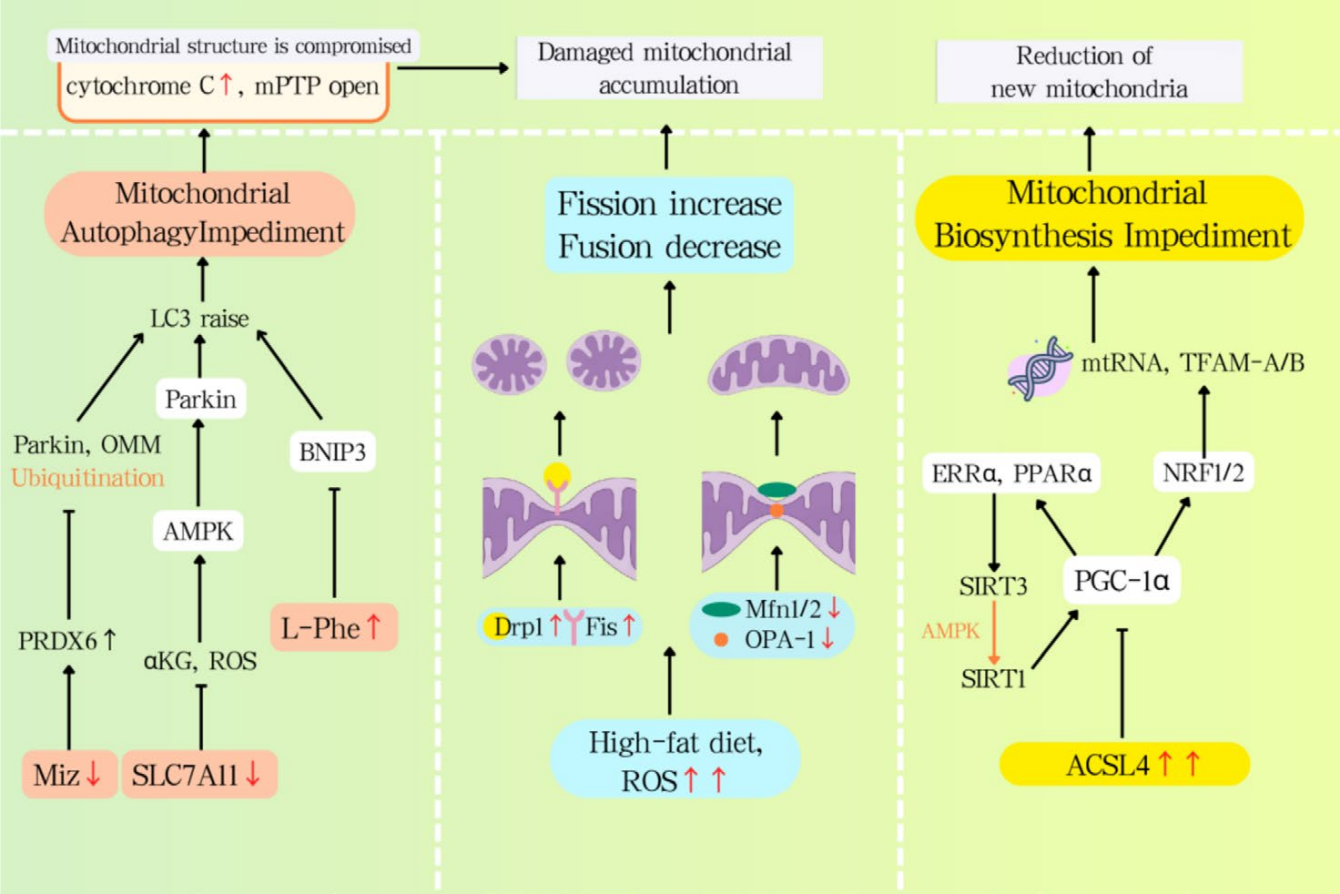
The effect of MASLD on the MQC process.

When mitochondria experience a loss of their electrical charge, they activate the PINK1/Parkin pathway. This, in turn, results in a rise in PINK1's presence on the outside layer of the mitochondria, which then triggers the phosphorylation of the Ser228/Ser402 spots. The activated PINK1 then phosphorylates the Ser65 site of ubiquitin, recruiting cytosolic Parkin proteins. This process ultimately facilitates the ubiquitination of outer mitochondrial membrane proteins, such as mitochondrial fusion protein 2 (Mfn2). Linker proteins like p62, NDP52, and OPTN bind to ubiquitinated substrates and LC3 proteins, thereby enhancing the formation and engulfment of autophagosomes. In the context of MASLD, a reduction in Miz1 triggers an increase in the levels of free Peroxiredoxin6 (PRDX6). This interaction with Parkin effectively prevents Parkin from ubiquitinating itself, let alone the outer mitochondrial membrane proteins (OMM), which ultimately hinders mitochondrial autophagy. This blockade speeds up the release of cytochrome c and prompts the opening of mitochondrial permeable transition pores (mPTPs). The AMPK signalling pathway has a significant impact on mitophagy in MASLD (Figure 1). The SLC7A11‐ROS/αKG‐AMPK axis in hepatocytes is crucial for controlling metabolic dysfunction‐associated steatohepatitis (MASH) progression, and Slc7a11 gene knockdown or knockout exacerbates steatohepatitis [1]. Moreover, Macrophage Stimulating 1 (Mst1) acts as a pivotal controller of mitophagy, downregulating Parkin levels through suppression of the AMPK signalling cascade. In MASLD, thereis an interactive relationship between mitochondrial autophagy and lipotoxicity; the reduced PINK1/Parkin expression compromises mitochondrial autophagy, accelerating cytochrome c release and mPTP opening. Consequently, lipotoxicity also stifles the mitochondrial autophagy pathway, including PINK1/Parkin and BNIP3/NIX, ultimately leading to the buildup of dysfunctional mitochondria. In both in vitro and in vivo models, the anthocyanin cation anthocyanin‐3‐O‐glucoside has been shown to bolster the condition of MASLD by enhancing PINK1‐mediated mitophagy [2] (Table 1).

**TABLE 1:** Drugs for the treatment of MASLD for different MQC stages.

Stage	Therapeutic drugs	Type of experiment	Function	Citation
Mitochondrial autophagy	Anthocyanin cation anthocyanin‐3‐O‐glucoside	In vitro and in vivo	Enhancing PINK1‐mediated mitochondrial autophagy.	[2]
	RNF31	In vivo	Promoting the degradation of p53 via ubiquitination, and promotes the expression of BNIP3.	[3]
	Polydatin	In vivo	Prompting SIRT1/SIRT3 activation, enhancing BNIP3‐induced mitophagy and reducing oxidative stress.	[4]
	USP10	In vitro	Activating the mTOR/Beclin1 signalling pathway and promotes autophagy flux.	[5]
	Liraglutide	In vivo	Increasing the ratio levels of autophagy proteins Beclin1 and LC3II/I.	[6]
Mitochondrial dynamics	Mdivi‐1	In vivo	Modulating AMPK signalling and Reducing Drp1.	[7]
	Metformin	In vivo	Modulating AMPK signalling.	[7]
	Rhein	In vitro and in vivo	Reducing the expression of Drp1.	[8]
Mitochondrial biogenesis	Deacetylase SIRT1	In vivo	Enhancing expression of PGC‐1α gene.	[9]
	Sofosbuvir (SOF)	In vivo	Enhancing expression of PGC‐1α and NRF1.	[10]
	Resmetirom (Rezdiffra)	In vivo	Activating of THR‐β promotes the gene expression related to the production of new mitochondria, mitochondrial autophagy, and the breakdown of fatty acids in hepatic cells.	[11]

#### 2.1.2 BNIP3/NIX Pathway

BNIP3, a classic mitophagy receptor located on the OMM, is activated by hypoxia in MASLD. Recent research has identified elevated levels of L‐phenylalanine (L‐Phe) in patients with MASLD, which reduces BNIP3 expression, thereby inhibiting BNIP3 from recruiting LC3B to form autophagosomes and initiating mitophagy to clear dysfunctional mitochondria (Figure 1). Serine 81 phosphorylation in NIX activates a mechanism that controls mitophagy during low‐oxygen conditions. Curiously, this process is connected to ferroptosis management; a lack of BNIP3/NIX boosts mitochondrial reactive oxygen species levels and the likelihood of ferroptosis [12]. RNF31, acting as an E3 ubiquitin ligase, encourages p53 breakdown via ubiquitination. When p53 levels drop, it gives a boost to BNIP3 expression, subsequently ramping up mitophagy in liver cells. Lab experiments show that boosting RNF31 in hepatocytes actually made mitophagy better, dialled down lipid buildup, and cut back on cell death [3]. Polydatin prompts SIRT1/SIRT3 activation, enhancing BNIP3‐induced mitophagy and reducing oxidative stress [4]. Given the protective role of ubiquitin‐specific protease (USP) in MASLD, therapies that target the deubiquitination aspect of the ubiquitin‐proteasome system have garnered considerable attention. For instance, USP10 has been found to boost the autophagic flux in MCDD‐fed mice and activate the mTOR/Beclin1 signalling pathway to alleviate fatty liver [5]. In a non‐autophagy dependent process, USP10 controls inflammation and lipid metabolism by negatively regulating Sirt6 (Table 1).

### 2.2 Mitochondrial Dynamics During MASLD

Mitochondrial morphology and function are dynamically regulated by fusion and fission:

#### 2.2.1 Fusion

OMM fusion requires mitofusins (Mfn1/Mfn2), Mfn2 tethers adjacent mitochondria via HR2 domain oligomerization, with Mfn1–Mfn2 heterocomplexes exhibiting superior GTP hydrolysis‐dependent efficiency. IMM fusion is mediated by OPA1 isoforms, which induce cristae remodelling and membrane curvature. MASLD downregulates Mfn2, impairing ER‐mitochondrial phosphatidylserine transfer and exacerbating ER stress. Reduced Mfn1/2 and OPA1 correlate with MASH severity (Figure 1).

#### 2.2.2 Fission

Mitochondrial fission occurs in response to oxidative stress‐induced damage, segregating impaired mitochondria from healthy ones. This process is regulated by Drp1 and its receptors, including FIS1, MFF, MID49 and MID51. Drp1 oligomerizes at OMM receptors, constricting mitochondria via GTP‐dependent helical contraction. Mdivi‐1 (a Drp1 inhibitor) and metformin normalise fission by modulating AMPK signalling [7]. Rhein inhibits Drp1, enhancing PINK1/Parkin mitophagy and reducing apoptosis [8] (Table 1).

### 2.3 Mitochondrial Biogenesis During MASLD

PGC‐1α, the coactivator for peroxisome proliferator‐activated receptor gamma, drives mitochondrial biogenesis by activating NRF1 and NRF2 as well as TFAM, thereby promoting mtDNA transcription and the import of nuclear‐encoded proteins. PGC‐1α also induces SIRT3 and PPARα, optimising oxidative phosphorylation. The long‐chain acyl‐CoA synthetase family member ACSL4 is highly expressed in liver tissue of patients with MASLD and is positively correlated with hepatocyte steatosis and fibrosis. Although ACSL4 does not directly participate in β‐oxidation, it inhibits PGC‐1α, thereby reducing β‐oxidation and ATP synthesis (Figure 1). Within the deacetylation process, SIRT1 activation plays a pivotal role in bolstering the expression of genes that contribute to the formation of mitochondria, including a boost in PGC‐1α levels. Sofosbuvir (SOF), a nucleotide analogue inhibitor of HCV polymerase, increases hepatic and muscular PGC‐1α and NRF1 expression in young female rats [10]. SGLT2 inhibitors are a novel class of oral antidiabetics that preserve mitochondrial function and enhance mitochondrial biogenesis. In 2024, the pharmaceutical world welcomed Rezdiffra, a groundbreaking medication, to the market for battling non‐alcoholic steatohepatitis in patients with moderate to severe fibrosis. This marvel operates by toggling on the THR‐β nuclear hormone receptor, which, in turn, fine‐tunes a plethora of genes. This process spurs the production of fresh mitochondria within liver cells, enhances mitochondrial autophagy, and facilitates the digestion of fatty acids [11] (Table 1).

## 3 MASLD Metabolic Stress State and MQC Dysregulation

MASLD encompasses various liver ailments, including MASH, liver cirrhosis, and hepatocellular carcinoma, which are separate from diabetic kidney disease and cardiovascular issues. As the development of MASLD advances, cardiovascular diseases have emerged as the primary cause of mortality among patients. Key risk factors associated with MASLD include obesity, insulin resistance, hypertension and elevated levels of triglycerides. Fatty liver changes can kickstart an increase in mitochondrial beta‐oxidation and reactive oxygen species production. This leads to dysfunctional mitochondrial quality control, triggering oxidative stress, inflammation and cell death—a whole host of pathological and physiological changes. Consequently, this accelerates the progression of MASLD into both MASH and liver fibrosis [13].

### 3.1 Oxidative Stress

**FIGURE 2:**
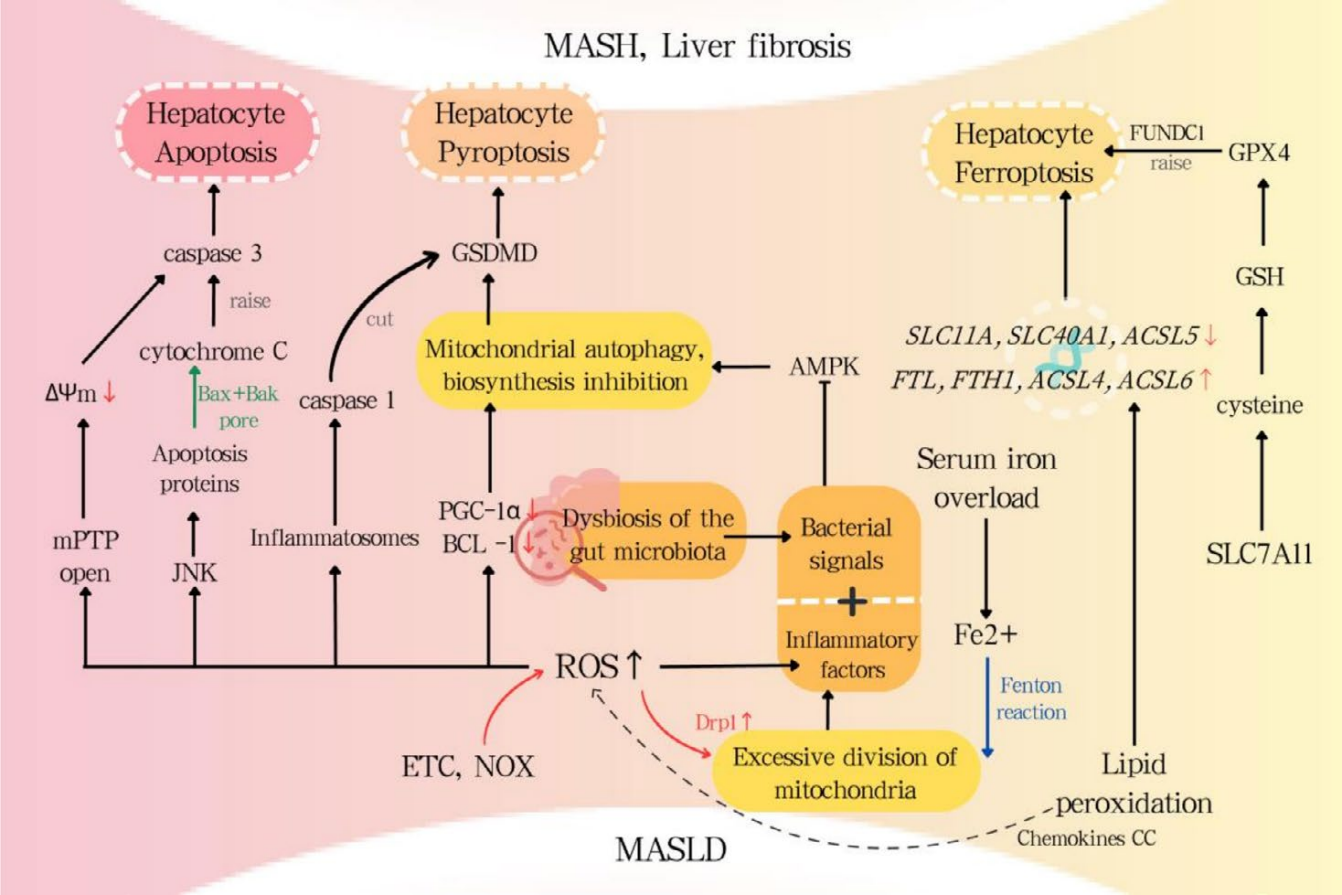
MASLD metabolic stress state and MQC dysregulation.

Oxidative stress pops up when there's an excess of ROS and the body's antioxidant defenses can't keep up. Lipid peroxidation products further upregulate hepatic CC chemokines during hepatocellular oxidative stress, promoting CCR5^+^ cell infiltration and myofibroblast activation, thereby driving fibrogenesis. Mitochondrial autophagy is crucial in regulating oxidative stress, and defects in mitochondrial autophagy may be a cause of oxidative stress. Experiments on mice have shown that oxidative stress during MASLD leads to reduced levels of PGC‐1α and Beclin‐1 within the body, which impairs mitophagy and biogenesis in liver cells (Figure 2). Experiments have demonstrated that ablation of the autophagy mediator PINK1 can induce hepatic lipid accumulation. In patients with MASLD, mitochondria exhibit characteristics of inefficient fatty acid metabolism and excessive ROS production. Although there's currently no data available regarding mitophagy biomarkers and NAFLD, data on mitochondrial quality indicates that mitophagy plays a role in the progression of the disease. Mouse experiments suggest that Asperuloside (ASP) activates NRF2 nuclear translocation and the NRF2/ARE pathway, thereby protecting MQC and enhancing MAFLD improvement [14].

### 3.2 Systemic Inflammatory State

Hepatic triglyceride accumulation, the hallmark of MASLD, stems from insulin resistance‐driven mobilisation of free fatty acids (FFAs) and de novo lipogenesis. Lipotoxic hepatocytes release damage‐associated molecular patterns (DAMPs; e.g., HMGB1) and pathogen‐associated molecular patterns (PAMPs), activating Kupffer cells (KCs) and recruiting infiltrating immune cells via pattern recognition receptors. NLRP3 inflammasome activation proteolytically cleaves pro‐IL‐1β and pro‐IL‐18, intensifying inflammation and hepatocellular injury. Mitochondrial DNA (mtDNA) released by damaged cells can promote the expression of inflammatory factors STING, NLRP3 and IL‐1β, collectively exacerbating inflammation. Exosomes play a pivotal role in how the liver processes sugars and fats, deals with inflammation, and develops chronic scarring.

Gut microbiota (GM) plays a crucial role in maintaining metabolic homeostasis; dysbiosis of the gut flora is widely recognised as a significant driver of the progression and development of MASLD. This connection stems from diverse processes: Gut dysbiosis compromises the intestinal barrier, enhancing permeability, thus facilitating fatty acid uptake. In MASH, bacterial signals and inflammatory cytokines inhibit AMPK, reducing mitochondrial biogenesis and mitophagy (Figure 2). In MASLD patients, levels of short‐chain fatty acids (SCFAs), beneficial metabolic compounds found in gut microbiota, are notably diminished; research indicates that short‐chain fatty acids (SCFAs) originating from symbiotic microbiota, such as acetates, modulate disease progression via specific receptors. Conversely, those suffering from MASLD typically have higher‐than‐normal amounts of ethanol, thanks to their gut microbiome. This surplus is likely due to certain bacteria that churn out ethanol, and it appears to go hand‐in‐hand with the advancement of the condition. While the role of gut microbiota in regulating MASLD has garnered extensive research attention as a novel therapeutic target, the precise relationship between specific microbial populations and the disease remains to be fully elucidated. This is crucial for understanding the impact and value of modulating gut microbiota and their metabolites as potential treatment strategies in the future.

### 3.3 Hepatocyte Death

The transition from MASLD to MASH, and from there to liver fibrosis and hepatocellular carcinoma, involves a highly intricate web of pathology. More than merely serving as a final destination, cell apoptosis is a pivotal force driving the disease forward. Chronic exposure to oxidative stress and inflammation takes a severe toll on the liver's mitochondria, which, after all, are the command central for both energy metabolism and determining whether a cell survives or dies [6]. The loss of mitochondrial transmembrane potential, coupled with a dip in the activity of mitochondrial respiratory chain complexes and a subsequent slump in ATP production, can throw the immune system out of whack. This, in turn, can kickstart liver cell death and put the brakes on liver regeneration following an injury [15]. Throughout the progression of MASLD, three closely associated forms of liver cell death that lead to fibrosis and apoptosis are necroptosis, pyroptosis and ferroptosis.

#### 3.3.1 Hepatocyte Apoptosis

Programmed cell death, is a fundamental mechanism that can be set off by internal or external cues. When it comes to the internal trigger, it's typically due to cellular stressors such as DNA damage and oxidative stress. This process relies heavily on the BCL‐2 protein family to manage the permeability of the outer mitochondrial membrane, effectively setting off the chain reaction of apoptosis. The extrinsic pathway kicks off when death receptors, like Fas, latch onto their ligands. This sets off a chain reaction, forming the death‐inducing signalling complex, or DISC, which, in turn, activates caspase‐8. Caspase‐8 then gets the effector caspases going, and they're the ones that actually carry out the apoptosis. Now, these two pathways aren't completely separate; they can interact. The extrinsic pathway can actually trigger the intrinsic pathway by cleaving Bid, effectively bringing it into the fold [16].

‘Two‐Hit Hypothesis’ of MAFLD posits initial lipid buildup followed by oxidative stress and inflammation, culminating in hepatic injury and apoptosis. Caspase‐3 activation and hepatic apoptosis are hallmark features of MASLD, correlating with the disease's severity. MQC gone awry also plays a role in hepatocyte apoptosis. When mitochondria undergo excessive fission and not enough fusion, leading to fragmentation, it can actually encourage cell death. Furthermore, in the absence of mitophagy, the PINK1/Parkin pathway can promote apoptosis through a BAX‐independent route (Figure 2). UPS‐Mediated Ubiquitination of OMM Drives Cytochrome C Release, Enhances Mitophagy, and Suppresses Apoptosis [17]. Research suggests that ginsenoside Rg5 shows promise in addressing MASLD in patients. It seems to do this by boosting Sirt1 protein expression, which in turn dials down the Notch1 signalling pathway. This modulation leads to improved lipid metabolism and appears to protect liver cells by reducing apoptosis. Rb1, another ginsenoside, mitigates gut microbiota‐driven inflammation and enhances mitochondrial quality control by modulating the DUSP‐1‐TMBIM‐6‐VDAC1 axis [18].

#### 3.3.2 Hepatocyte Pyroptosis

Pyroptosis, a type of programmed cell death in hepatocytes, is closely tied to inflammasomes in MASH. This process hinges on caspase activation, which then unleashes a cascade of inflammatory responses. There are basically two routes to get this show on the road. The first, known as the canonical pathway, relies on inflammasomes like NLRP1, NLRP3, NLRC4 and AIM2 to get caspase‐1 fired up. This then cleaves Gasdermin D (GSDMD), forming pores that leak cellular goo, ultimately leading to the cell's demise. The second path, the non‐canonical one, uses caspases‐4/5/11 to activate the inflammasome and directly chop up GSDMD, kicking off the pyroptotic process [16]. Both methods are intricately linked. When caspase‐4/5/11 are activated, they stimulate Pannexin‐1 channels, which in turn release K+ into the extracellular space. This action sparks the activation of the NLRP3 inflammatory body, leading to the induction of caspase‐1's activation, which subsequently activates the classical pathway. ROS also play a significant role in the execution of pyroptosis. An overabundance of ROS can trigger excessive mitochondrial fission, a process mediated by Drp1. This, in turn, ramps up the activation of caspase‐1 and the NLRP3 inflammasome, ultimately leading to pyroptosis (Figure 2). Dysfunctional mitophagy triggers ROS‐mediated GSDMD oligomerization via C192 oxidation, culminating in membrane rupture and pyroptosis. Moreover, studies have shown that various long non‐coding RNAs, like GAS5, as well as specific natural extracts such as ginsenoside Re, have a substantial impact on curbing NLRP3‐mediated pyroptosis, which is a crucial process that drives the advancement of MASLD. These factors may soon become a prime therapeutic focus [19].

#### 3.3.3 Hepatocyte Ferroptosis

Ferroptosis, an iron‐dependent cell death mechanism, is distinguished by iron excess, lipid peroxidation, ROS accrual, and plasma membrane rupture. In MASLD patients, serum iron levels are 1.5 times the average, with ferroptosis resulting from liver lipid peroxidation being the initial form of cellular death.

In the context of MASLD and MASH, the mechanisms behind ferroptosis involve three primary pathways. First off, an imbalance in iron homeostasis results in an overload of iron ions, which, through the Fenton reaction, produces a surge of hydroxyl radicals. This sudden spike sets off a chain reaction of oxidative stress, culminating in the devastation of cellular nuclei, membranes, organelles, and proteins. Moreover, lipid metabolism is initiated by lipid peroxidation, which ultimately results in ferroptosis. Lastly, the disruption of the SLC7A11‐GSH‐GPX4 axis causes a massive depletion of the antioxidant GSH by lipid peroxides and hydroperoxides, rendering GPX4 ineffective (Figure 2). In liver fibrosis, FUNDC1‐dependent GPx4 mitophagy enhances ferroptosis. In the genetic domain, the grading of liver steatosis correlates with eight iron death‐related genes, namely *ACSL3*, *ACSL4*, *AKR1C1*, *AKR1C2*, *CS*, *FADS2*, *GSS* and *PGD*; in MASH patients, the expression levels of iron death‐related genes including *SLC11A2*, *CP*, *SLC40A1* and *ACSL5* are decreased, whereas the expression levels of *FTL*, *FTH1*, *ACSL4* and *ACSL6* are increased (Figure 2).

Mitochondrial quality control plays a significant role in ferroptosis associated with MASLD. NRF2 is key in maintaining mitochondrial health. NRF2 can ramp up the protein levels of Mfn2, encouraging mitochondrial fusion and putting the brakes on fission. Mitochondrial ROS and ATP play a pivotal role in modulating ferroptosis. When OPA1 encourages mitochondrial fusion, the resulting accumulation of ROS and fatty acids can kickstart lipid peroxidation, ultimately triggering ferroptosis. Mitochondrial autophagy plays a complex, double‐edged role in the regulation of ferroptosis. On one hand, damaged or dysfunctional mitochondria can unleash a torrent of ROS and pro‐apoptotic factors, essentially jump‐starting the ferroptosis process. On the other hand, when mitophagy kicks in to clear away these problematic mitochondria, it puts the brakes on ferroptosis, effectively keeping it in check. Targeting ferroptosis, besides liraglutide being able to inhibit ferroptosis by activating AMPK/ACC, recent data demonstrate that FerroTerminator1 alleviates MASH by inhibiting Acsl4 expression, thereby reducing lipid peroxidation [20].

## 4 Conclusion

This letter frames MQC as a central driver in the development of MASLD. When mitochondrial autophagy, turnover, and biogenesis go awry, the result is a cascade of hepatic lipotoxicity, oxidative stress, and inflammatory responses. Importantly, a breakdown in MQC speeds the transition from simple steatosis to MASH and liver fibrosis by heightening metabolic strain, provoking inflammation, and promoting cell death. New therapeutic approaches show potential for reestablishing mitochondrial equilibrium in MASLD by targeting the MQC network. Looking ahead, research should emphasise clinical testing of MQC‐oriented treatments and deepen our understanding of how the gut–liver axis influences mitochondrial function. Framing MQC as a pivotal therapeutic frontier could stop MASLD in its tracks by interrupting its progression.

## Author Contributions

X.C. and M.M. designed the research study. Z.G. and S.O. carried out supervision work. Y.G. provided help and advice. H.Z. wrote, reviewed and edited. W.F. drafted the manuscript and completed the figures. J.W. and N.L. drafted the manuscript. All authors contributed to editorial changes in the manuscript. All authors read and approved the final manuscript.

## Conflicts of Interest

The authors declare no conflicts of interest.

## Data Availability

Data sharing not applicable to this article as no datasets were generated or analysed during the current study.

## References

[1] T. Lv , X. Fan , C. He , et al., “SLC7A11‐ROS/αKG‐AMPK Axis Regulates Liver Inflammation Through Mitophagy and Impairs Liver Fibrosis and NASH Progression,” Redox Biology 72 (2024): 103159, 10.1016/j.redox.2024.103159. 38642501 PMC11047786

[2] K. A. Dias , L. A. Oliveira , S. M. S. Pereira , et al., “Anti‐Inflammatory and Antioxidant Effects of Anthocyanins in Nonalcoholic Fatty Liver Disease (NAFLD): A Systematic Review of In Vivo Studies,” Critical Reviews in Food Science and Nutrition (2025): 1–18, 10.1080/10408398.2025.2472882. 40045715

[3] Y. Chen , F. Yang , Y. Shi , et al., “RNF31 Alleviates Liver Steatosis by Promoting p53/BNIP3‐Related Mitophagy in Hepatocytes,” Free Radical Biology & Medicine 219 (2024): 163–179, 10.1016/j.freeradbiomed.2024.04.214. 38615890

[4] J. He , Y. C. Qian , Y. C. Yin , J. R. Kang , and T. R. Pan , “Polydatin: A Potential NAFLD Therapeutic Drug That Regulates Mitochondrial Autophagy Through SIRT3‐FOXO3‐BNIP3 and PINK1‐PRKN Mechanisms: A Network Pharmacology and Experimental Investigation,” Chemico‐Biological Interactions 398 (2024): 111110, 10.1016/j.cbi.2024.111110. 38876248

[5] S. L. Xin , X. L. Pan , X. Y. Xu , and Y. Y. Yu , “USP10 Alleviates Palmitic Acid‐Induced Steatosis Through Autophagy in HepG2 Cells,” Journal of Clinical and Translational Hepatology 11, no. 1 (2023): 45–57, 10.14218/JCTH.2022.00060. 36406315 PMC9647103

[6] W. Tong , L. Ju , M. Qiu , et al., “Liraglutide Ameliorates Non‐Alcoholic Fatty Liver Disease by Enhancing Mitochondrial Architecture and Promoting Autophagy Through the SIRT1/SIRT3‐FOXO3a Pathway,” Hepatology Research 46, no. 9 (2016): 933–943, 10.1111/hepr.12634. 26666995

[7] M. Elbadawy , K. Tanabe , H. Yamamoto , et al., “Evaluation of the Efficacy of Mitochondrial Fission Inhibitor (Mdivi‐1) Using Non‐Alcoholic Steatohepatitis (NASH) Liver Organoids,” Frontiers in Pharmacology 14 (2023): 1243258, 10.3389/fphar.2023.1243258. 37900170 PMC10600465

[8] H. Li , Y. Jia , D. Yao , M. Gao , L. Wang , and J. Liu , “Rhein Alleviates Myocardial Ischemic Injury by Inhibiting Mitochondrial Division, Activating Mitochondrial Autophagy and Suppressing Myocardial Cell Apoptosis Through the Drp1/Pink1/Parkin Pathway,” Molecular Biology Reports 51, no. 1 (2024): 266, 10.1007/s11033-023-09154-1. 38302764

[9] J. Liao , X. Xie , N. Wang , et al., “Formononetin Promotes Fatty Acid β‐Oxidation to Treat Non‐Alcoholic Steatohepatitis Through SIRT1/PGC‐1α/PPARα Pathway,” Phytomedicine 124 (2024): 155285, 10.1016/j.phymed.2023.155285. 38185065

[10] H. A. Hafez , A. M. Atoom , R. H. M. Khafaga , et al., “Direct‐Acting Antiviral Drug Modulates the Mitochondrial Biogenesis in Different Tissues of Young Female Rats,” International Journal of Molecular Sciences 24, no. 21 (2023): 15844, 10.3390/ijms242115844. 37958828 PMC10647297

[11] S. A. Harrison , P. Bedossa , C. D. Guy , et al., “A Phase 3, Randomized, Controlled Trial of Resmetirom in NASH With Liver Fibrosis,” New England Journal of Medicine 390, no. 6 (2024): 497–509, 10.1056/NEJMoa2309000. 38324483

[12] S. I. Yamashita , Y. Sugiura , Y. Matsuoka , et al., “Mitophagy Mediated by BNIP3 and NIX Protects Against Ferroptosis by Downregulating Mitochondrial Reactive Oxygen Species,” Cell Death and Differentiation 31, no. 5 (2024): 651–661, 10.1038/s41418-024-01280-y. 38519771 PMC11094013

[13] M. M. Syed‐Abdul , “Lipid Metabolism in Metabolic‐Associated Steatotic Liver Disease (MASLD),” Metabolites 14, no. 1 (2023): 12, 10.3390/metabo14010012. 38248815 PMC10818604

[14] C. He , Q. Zhang , R. Zhu , G. Tse , and W. T. Wong , “Asperuloside Activates Hepatic NRF2 Signaling to Stimulate Mitochondrial Metabolism and Restore Lipid Homeostasis in High Fat Diet‐Induced MAFLD,” European Journal of Pharmacology 983 (2024): 177003, 10.1016/j.ejphar.2024.177003. 39278309

[15] G. G. Lamanilao , M. Dogan , P. S. Patel , et al., “Key Hepatoprotective Roles of Mitochondria in Liver Regeneration,” American Journal of Physiology. Gastrointestinal and Liver Physiology 324, no. 3 (2023): G207–G218, 10.1152/ajpgi.00220.2022. 36648139 PMC9988520

[16] K. Cai , H. Jiang , Y. Zou , et al., “Programmed Death of Cardiomyocytes in Cardiovascular Disease and New Therapeutic Approaches,” Pharmacological Research 206 (2024): 107281, 10.1016/j.phrs.2024.107281. 38942341

[17] G. Quarato , L. Mari , N. J. Barrows , et al., “Mitophagy Restricts BAX/BAK‐Independent, Parkin‐Mediated Apoptosis,” Science Advances 9, no. 21 (2023): eadg8156, 10.1126/sciadv.adg8156. 37224250 PMC10208567

[18] X. Pu , Q. Zhang , J. Liu , et al., “Ginsenoside Rb1 Ameliorates Heart Failure Through DUSP‐1‐TMBIM‐6‐Mediated Mitochondrial Quality Control and Gut Flora Interactions,” Phytomedicine 132 (2024): 155880, 10.1016/j.phymed.2024.155880. 39053246

[19] J. Lin , H. Wang , R. Zhao , et al., “Ginsenoside re Ameliorates Thioacetamide‐Induced Acute Liver Injury Through Inhibiting Autophagy‐NLRP3 Inflammasome Pathway,” Frontiers in Pharmacology 16 (2025): 1592203, 10.3389/fphar.2025.1592203. 40620672 PMC12226568

[20] L. Tao , X. Yang , C. Ge , et al., “Integrative Clinical and Preclinical Studies Identify FerroTerminator1 as a Potent Therapeutic Drug for MASH,” Cell Metabolism 36, no. 10 (2024): 2190–2206.e5, 10.1016/j.cmet.2024.07.013. 39142286

